# Increasing the Complexity of the Illumination May Reduce Gloss Constancy

**DOI:** 10.1177/2041669517740369

**Published:** 2017-12-09

**Authors:** Gunnar Wendt, Franz Faul

**Affiliations:** Institut für Psychologie, Universität Kiel, Germany

**Keywords:** light, gloss perception, material perception, gloss constancy

## Abstract

We examined in which way gradual changes in the geometric structure of the illumination affect the perceived glossiness of a surface. The test stimuli were computer-generated three-dimensional scenes with a single test object that was illuminated by three point light sources, whose relative positions in space were systematically varied. In the first experiment, the subjects were asked to adjust the microscale smoothness of a match object illuminated by a single light source such that it has the same perceived glossiness as the test stimulus. We found that small changes in the structure of the light field can induce dramatic changes in perceived glossiness and that this effect is modulated by the microscale smoothness of the test object. The results of a second experiment indicate that the degree of overlap of nearby highlights plays a major role in this effect: Whenever the degree of overlap in a group of highlights is so large that they perceptually merge into a single highlight, the glossiness of the surface is systematically underestimated. In addition, we examined the predictability of the smoothness settings by a linear model that is based on a set of four different global image statistics.

## Introduction

During the past two decades, considerable progress has been made in identifying cues in the proximal stimulus that are used by the visual system to infer the material properties of objects (Fleming, 2014) and this is particularly true for the special case of gloss perception (Chadwick & Kentridge, 2015). In the present study, we investigate gloss perception with a focus on highlights in the proximal stimulus that are known to be used by the visual system to judge the glossiness of a surface.

**Figure 1.**
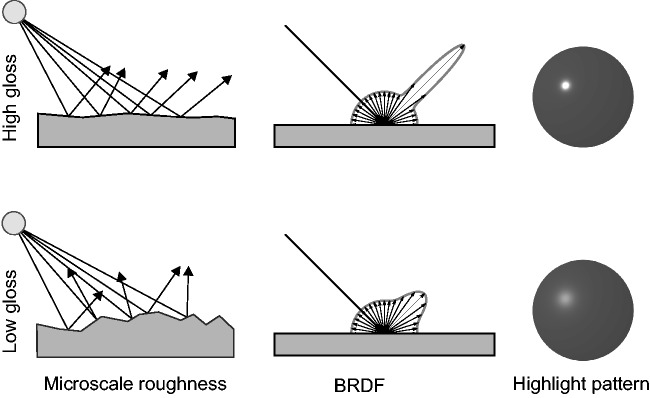
Microfacet models (Cook & Torrance, 1982) consider surfaces as composed of tiny mirrors (microfacets) with varying orientation. The degree of specular reflection of a surface is determined by the orientation distribution of these microfacets (left column): High gloss results from smooth surfaces whose microfacets have very similar orientations (top left) and low gloss from rough surfaces whose microfacets differ more strongly in orientations (bottom left). On a broader scale, surface reflectance can be described by a BRDF (middle column). The BRDF of an isotropic material describes the spatial distribution of the reflected light for each angle of the incident light that hits the material. The hemispherical part of the BRDF in the middle column represents the diffuse component, which is assumed to reflect incoming light equally in all directions. The specular component, on the other hand, is directionally selective and is represented by the “specular lobe.” The exact shape of the specular lobe depends on the microscale roughness, which in turn determines properties and appearance of the highlights (right column). BRDF = bidirectional reflectance distribution function.

Highlights are related to surface locations from which the incident light is specularly reflected to the eyes of an observer and their properties depend mainly on surface reflectance, object shape, and the lighting conditions. In the context of material perception, the relationship between highlight properties and physical properties of surfaces is of primary interest. Most models of glossy materials distinguish two additive components, namely, diffuse and specular reflectance (see Figure 1). Diffuse reflection is usually based on Lambert’s law. The core assumption is that the light is reflected in all directions with an intensity that depends on the angle between the direction of the incident light and the orientation of the surface normal. The specular component that is associated with the glossiness of a surface is often described by a microfacet model. In this model, the surface is assumed to be composed of tiny flat mirror elements, the microfacets, whose sizes are similar to the wavelength of light. The amount of specular reflection is determined by the roughness of the surface, which is directly related to the distribution of the microfacets’ orientation (see Figure 1, first column). The surface reflectance properties can formally be described by a bidirectional reflectance distribution function (or BRDF; see Nicodemus, Richmond, Hsia, Ginsberg, & Limperis, 1977) that gives the ratio of outgoing radiance to incoming irradiance for each possible pair of incoming and outgoing directions. The second column in Figure 1 illustrates, in schematic form, typical BRDFs of glossy surfaces. Surface reflectance strongly affects the properties of highlights in the proximal stimulus (Figure 1, third column): Surfaces with a very smooth microscale structure reflect the incident light almost exclusively in one dominant direction, and if the line of sight is oriented in this particular direction, a small, sharp, and relatively bright highlight is seen at the fixation point. With increasing roughness, the highlight becomes larger, blurrier, and less intensive.

Numerous studies suggest that the visual system uses this correlation between surface reflectance and highlight properties as a cue to ascribe the subjective material quality of glossiness to a surface (e.g., Beck & Prazdny, 1981; Forbus, 1977; Leloup, Pointer, Dutré, & Hanselaer, 2010; Marlow & Anderson, 2013; Marlow, Kim, & Anderson, 2011, 2012; Qi, Chantler, Siebert, & Dong, 2014, 2015; Wendt, Faul, & Mausfeld, 2008). However, the interpretation of highlights is complicated by the fact that their properties depend also on factors not related to the material, such as surface shape and the lighting conditions. To the extent that glossiness is understood as a subjective correlate of material properties, these additional influences on highlights can be regarded as interfering factors and this leads to the question to what extent they also influence perceived glossiness.

**Figure 2.**
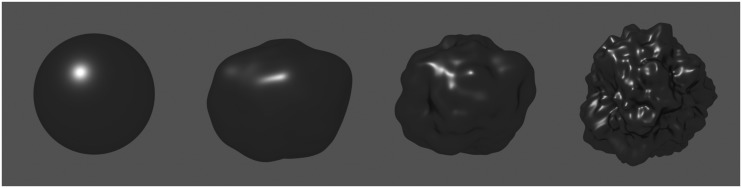
Four objects of different shape were rendered using identical reflection properties and the same point light source. The differences in the three-dimensional structure lead to different highlight patterns and also affect the perceived glossiness of the objects.

Figure 2 illustrates the influence of surface shape on perceived glossiness. All four objects have an identical BRDF and were rendered under the same illumination (a single point light source). From left to right, the three-dimensional (3D) structure of the objects becomes more and more ragged on a mesoscale. In the simplest case of a perfect sphere, the local curvature is constant across the surface and all surface normals have a unique orientation. A point light source will therefore always generate a single highlight with a circular shape. The other depicted objects show a greater variety of local curvatures and in general contain several areas with similar orientations of the surface normals. Accordingly, the proximal stimulus is more complex and contains several highlights varying in number, size, and shape. As a rule, the highlights become smaller and less blurry with increasing local curvature at the corresponding surface point.

These changes in the spatial properties of the highlights lead also to changes in the perceived glossiness of the surface. For instance, the rightmost object in Figure 2 appears considerably glossier than the sphere on the left. This indicates that the visual system does not fully discount the effect of shape on the highlight pattern and, hence, fails to provide perfect gloss constancy. Although some studies have found that the degree of gloss constancy under changes in shape can be improved by enriching the proximal stimulus with additional information from motion, disparity, or color (cf. Sakano & Ando, 2010; Wendt, Faul, Ekroll, & Mausfeld, 2010), perfect constancy is seldom achieved, that is, two surfaces with identical BRDF but different 3D structures usually differ in perceived glossiness (Nishida & Shinya, 1998; Vangorp, Laurijssen, & Dutré, 2007).

That the highlight pattern in the proximal stimulus also depends on the lighting conditions is not surprising, because highlights are essentially mirror images of light sources. The highlight pattern produced by a given surface illuminated by a single point light source, for instance, differs in general from the one resulting under more complex lighting conditions, like configurations with multiple light sources or realistic light fields that include interreflections of the environment.

A number of empirical studies have found that the structure of the illumination may also strongly influence perceived glossiness (Doerschner, Boyaci, & Maloney, 2010; Fleming, Dror, & Adelson, 2003; Motoyoshi & Matoba, 2012; Olkkonen & Brainard, 2010, 2011; Pont & te Pas, 2006). The light fields that were used in these experiments varied in complexity and structure. Pont and te Pas (2006), for instance, used comparatively simple lightings, such as collimating light from various directions, hemispherical diffuse, and fully diffuse light, to illuminate computer-generated spheres that were rendered using four different BRDFs. They found that observers could not reliably distinguish between different materials: Spheres with equal BRDFs were often perceived to be of different material when rendered under a different lighting and, conversely, objects with different BRDFs and different illuminations often appeared to be made of the same material. Fleming et al. (2003) compared the effects of realistic and artificial illuminations on gloss perception and gloss constancy. Their subjects adjusted the reflectance parameters of a sphere under a fixed standard illumination so that its perceived glossiness matched that of a sphere of fixed material rendered in a test illumination. The test illuminations used by the authors comprised a number of real-world illumination maps, that is, spherical panoramas from a variety of indoor and outdoor scenes, as well as artificial illuminations, such as single and multiple point light sources, and different noise patterns. They found that the matching performance was strongly influenced by the lighting condition. The matching errors, measured as difference in the objective reflectance parameters of test and match object, were considerably higher under artificial than under real-world illumination.

### Aim of the Present Study

In most of these previous studies, glossiness estimates were compared within or between certain classes or categories of illuminations, like simple versus complex or realistic versus artificial lights. The aim of the present study is to test, in which way gloss perception is affected when the lighting changes gradually between simple and complex lighting. To this end, we created computer-generated scenes, where an object was illuminated by three point light sources whose relative positions in space were systematically varied. Starting with a simple lighting condition, where all three point lights had identical positions (which is equivalent to a single point light source), we gradually increased the distance or spread between these lights and in this way created a complex illumination comprising multiple point light sources.

The changes that were applied to the light field influenced the highlight patterns of the stimuli in several ways: Since each highlight on a surface is associated with one particular point light source, the total number of highlights varied between the one point light situation and the three point lights situation. Furthermore, depending on the size of the light spread and the microscale roughness of the surface, intermediate states occurred, where different highlights overlapped and merged into one spatially extended highlight. We were interested in how the visual system deals with such ambiguous stimulus situations. From what we have learned so far about how the visual system estimates glossiness based on highlight patterns, it is likely that the visual system interprets an extended highlight as being caused by a less glossy surface. In this case, the visual system would underestimate the glossiness of the surface and thus fail to achieve perfect gloss constancy.

**Figure 3.**
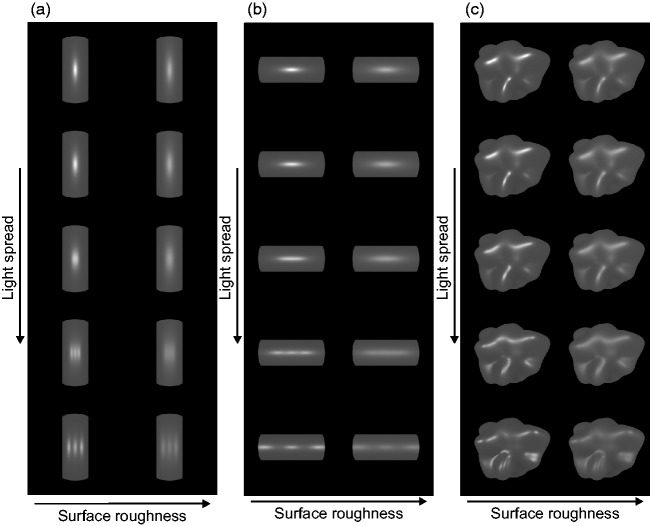
(a–c) Each of the three panels shows two columns of objects with identical shapes. In the first column, the objects have a lower microscale roughness than in the second column. From top to bottom, the light spread is gradually increased (cf. Figure 4). The resulting highlight patterns seem to affect gloss perception differently, depending on the relationship between the direction of highlight variability and the objects’ local curvature. For example, the effect on perceived glossiness seems more pronounced in (a), where the light-source spread affects the width of the (merged) highlight along the direction with nonzero curvature, than in (b), where it leads to an elongation of the highlight in the direction of zero curvature.

**Figure 4.**
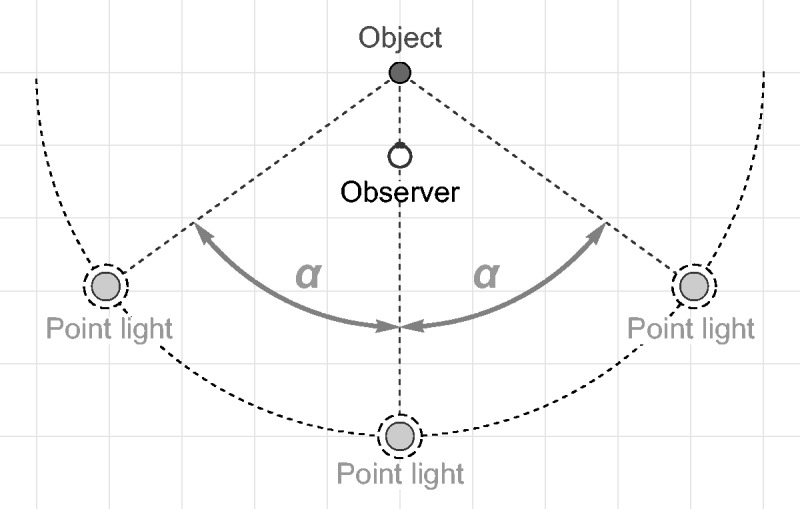
The general layout of our test scenes (view from top): All scene elements, that is, the test object, the three point lights, and the cameras were placed in the same horizontal plane. The test object was located at the center of the scene, the cameras representing the two eyes of the observer were placed at (−0.03, −1.0) and (0.03, −1.0), respectively, where the coordinates refer to relative units. One of the three point light sources was always located behind the observer at (0.0, −5.0), the locations of the two remaining point lights, which always had a constant distance of 5 units to the center of the object, were determined by the light spread parameter α.

Informal observations with a simple geometric object confirmed that such effects can indeed occur: The two columns in Figure 3(a) show an array of cylinders with identical surface roughness, with roughness increasing from left to right. From top to bottom, the spread between three point light sources (that are all positioned in a horizontal plane on a circular arc, i.e., with the same distance to the object, see Figure 4) is gradually increased. The highlights seen at the top (angle = 0) first widen in the horizontal direction, in which the cylinder has a convex curvature, and the glossiness impression gets correspondingly weaker. For even higher spreads, the merged highlights eventually split up into three individual ones. The effect of this split-up on perceived glossiness seems somewhat elusive, though. At least under monocular viewing conditions, this minutely arranged highlight pattern appears more like a texture than as highlight and thus fails to evoke a clear impression of gloss (see also Van Assen, Wijntjes, & Pont, 2016). A clearly different effect can be observed in Figure 3(b), where the highlight pattern varies along the direction of zero curvature of the cylinder: Again, with increasing light spread, the width of the highlight increases until it splits up into three separate highlights. However, in this case, the perceived glossiness seems hardly affected by these changes in the highlight configuration.

This indicates that the effect of increasing the distance between point lights depends on the relationship between the direction of (local) highlight pattern variability and the local curvature of the surface. It is thus not clear, how this manipulation will influence the perceived glossiness of surfaces with more complex 3D geometries—that is, surfaces with a variety of different curvatures with different orientations (see, for instance, Figure 3(c)). In these cases, the highlight patterns will contain different local groups of highlights, where each of them may affect the gloss impression to a different degree. Several ways are imaginable, how the visual system reaches an overall estimate of the glossiness of such a surface. For instance, it could integrate the different local signals in one way or another, or it could regard a certain highlight group as representative for the glossiness of the entire surface. To explore a potential interaction with the 3D structure of the surface, we also varied the shape of the test objects in our experiments.

## Experiment 1

### Stimuli

To test whether such changes in the lighting conditions affect the glossiness perception of surfaces with complex 3D shapes, we used computer-generated stimuli. Each test stimulus consisted of a 3D surface with certain reflection properties that was illuminated by three point light sources (see Figure 4). The game engine Unity (version 5.3.4) was used for the construction and the display of our scenes, and the control of the experiment.

### Surfaces

**Figure 5.**
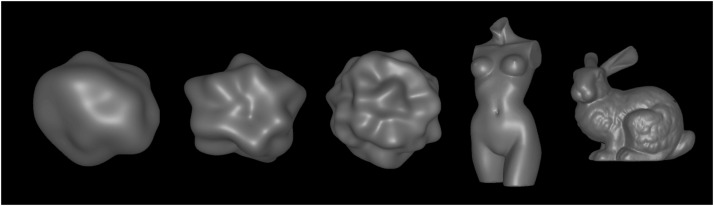
The five different shapes used as test objects in our experiments. From left to right: blob#1, blob#2, blob#3, statue, and bunny.

The test objects had one of five different shapes (see Figure 5). Three of them were generated with the 3D software blender (version 2.76), another one (the “statue”) was downloaded from a free 3D object database (download link: www.archive3d.net/? a=download&id=c3ba8f71), and the fifth was the well-known Stanford bunny (“bunny”). The three objects made with blender had blob-like shapes with different spatial frequencies. We will refer to these objects as “blob#1,” “blob#2,” and “blob#3,” respectively. The basic mesh was an icosphere with a resolution of six subdivisions, that is, it consisted of 20,480 triangles. To change the objects’ mesoscale shape, we applied the “displace” modifier, using a three-dimensional clouds texture based on improved Perlin noise (with default settings “grayscale” and “soft”). The “strength” parameter of the modifier had the values 1.0 for blob#1 and blob#2 and 0.5 for blob#3. The “size” parameter of the texture was 1.0 for blob#1, 0.7 for blob#2, and 0.4 for blob#3. The “depth” and “nabla” parameters for the texture were held constant for all three objects with values of 0 and 0.03, respectively.

The statue object consisted of 14,882 triangles, the bunny of 55,051 triangles (which is lower than in the original Stanford bunny; we used this lower resolution mesh, since in Unity the number of triangles in a single mesh must not exceed 65,535). The shading of all five objects was set to smooth and they were scaled equally in all dimensions to roughly have similar sizes (see Figure 5): The maximum vertical extensions of the objects were 4.2, 4.0, and 4.3 degrees of visual angle (dva) for blob#1, blob#2, and blob#3, respectively, 6.3 dva for the statue, and 4.2 dva for the bunny.

### Material Properties

To simulate surface reflectance, we used the built-in standard shader of Unity, which meets basic physical principles, such as energy conservation, and also includes Fresnel reflections. We used a constant grayish color (rgb = 0.5, 0.5, 0.5) for the diffuse component (albedo). It is worth mentioning that Unity uses the Disney implementation of the diffuse component rather than Lambert’s law. The Disney diffuse shading is based on empirical measurements and is modeled according to a microfacet approach (Burley, 2012). For the specular component, the GGX model (Walter, Marschner, Li, & Torrance, 2007) is used. However, instead of the original roughness parameter, Unity uses a smoothness scale to control the blurring of the specular reflections (the relationship is *roughness = *(1*−smoothness*)^2^). In our experiments, the subjects adjusted a scaled version of the smoothness parameter, in which equal scale distances approximately correspond to equal steps in perceived glossiness (see Appendix A). In the following, whenever we refer to the smoothness parameter, we mean this scaled smoothness scale. The metallic parameter of the specular component, which is mainly used to determine the color of the highlights, was set to 0, so that the highlights appeared in the color of the lights.

### Lighting

The general layout of our scenes is shown in Figure 4: All elements of the scene were located in the same horizontal plane. The three point light sources always had a constant distance of 5 units to the object, which was placed in the center of the scene. One point light was always located at a fixed position, namely, in front of the test object (the point light at the center bottom in Figure 4). The positions of the two remaining lights were determined by a parameter that we will refer to as the “light spread α.” In our experiments, the light spread parameter had values between 0 and 1, where a value of 0 means that all lights are located at the same central position and a value of 1 means an angle of 90° between the central point light and the left or the right point light, respectively.

The color of the point lights was set to white (rgb = 1.0, 1.0, 1.0) and the intensity was chosen to be either 0.5 or 1.5. The maximum effective range of the lights, that is, the distance beyond which the intensity drops to zero, was set to 10 units (see Appendix B for a more detailed description). The remaining parameters of the light components were set to the default values, including the usage of soft shadows (however, due to our scene arrangement, self-shadowing effects were visually negligible). In the settings for global environment lighting, we disabled the “skybox” and “sun” options. Instead, we used an ambient source with a constant white color (rgb = 1.0, 1.0, 1.0) at an intensity level of 0.6.

### Apparatus and Viewing Conditions

Our stimuli were displayed on a TFT monitor (EIZO CG243W) with a resolution of 1920 by 1200 pixels at a screen width of 52 cm and a screen height of 32.5 cm. The CIE 1931 color coordinates of maximum white (rgb = 1.0, 1.0, 1.0) were *x* = 0.313 and *y* = 0.327 at a luminance *Y* = 122.57 cd/m^2^. In order to enhance the impression of three dimensionality as well as the perception of glossiness (Sakano & Ando, 2010; Wendt et al., 2010; Wendt et al., 2008), we presented our stimuli stereoscopically, using a mirror stereoscope (ScreenScope) that was mounted on the monitor. The total path length of the light between the monitor screen and the observer’s eyes was 50 cm. For a stereoscopic set-up, we had to use two different camera components for each scene in Unity that captured the images for the two eyes of the observer from different positions. The exact locations of the two cameras were (−0.03, −1.0) for the left eye and (0.03, −1.0) for the right eye, respectively, so the observer was about 1 unit away from the center of the object (see Figure 4). As further settings for the camera components, we used perspective projection and some starting values for the near and far clipping plane (0.5 and 3.0 units, respectively) as well as for the field of view (60°). However, these values were changed by a script when the experiment was started, because we implemented the so-called off-axis perspective projection, as described in Kooima (2008).

For each scene, the two monocular half-images that were taken by the corresponding pair of cameras were displayed side by side on the monitor screen with no gap between them. Each image had a width of 30% of the width of the monitor screen and a height of 50% of the screen’s height. We used a solid blueish background color (rgb = 0.192, 0.302, 0.475) for all cameras.

### Procedure

Before we conducted our main experiment, we tested in a preexperiment (see Appendix C), whether the glossiness settings of our subjects depend on the psychophysical method (see Doerschner et al., 2010). We compared a pair comparison task in a staircase procedure with a matching task and actually found a statistically significant main effect of the experimental method. However, since we could not detect any systematic method-dependent deviations in the general trends and the effect sizes, we decided to use the matching procedure in our experiment, because it was significantly less time-consuming and exhausting than the other method.

The matching object was always blob#2 (see Figure 5), whose smoothness parameter could be interactively manipulated by the subjects with the left and right arrow keys of the keyboard. The subjects were asked to adjust the glossiness of the match stimulus so that it appeared indistinguishable from the glossiness of the test stimulus. To make it more difficult for the subjects to simply match local highlight features, we presented the match object dynamically, by rotating it counterclockwise around its vertical middle axis at a speed of 60°/s. The spatial arrangement of the match scene was identical to that of the test scene (see Figure 4), with the exception that we used only one point light source with a fixed location in front of the object (see the center bottom light in Figure 4) and an intensity of 1.5. The test stimulus was always presented statically. In each trial, the two half-images of the match stimulus were presented on the top half of the monitor screen, while the test stimulus always appeared on the bottom half.

In total, 350 different conditions were realized: We examined five scaled smoothness levels (0.2, 0.3, 0.4, 0.5, and 0.6), seven levels of the light spread parameter α (0.0, 0.04, 0.8, 0.12, 0.16, 0.32, and 0.6), five shapes (see Figure 5), and two different levels for the intensity of the test point lights (0.5 and 1.5). Each condition was tested 4 times. The entire set of 1,400 test stimuli was presented in random order during the experiment. In a small text field located at the center between the match and the test stimulus, the current trial number and the total number of trials were shown, to provide constant feedback about the progress of the experiment. To complete a match, the subject pressed the space bar on the keyboard. The next trial started after a short pause of 1 second, during which only the text field and the blueish background color were visible. There was no time limit imposed for the matching task and the subjects were allowed to interrupt a session at any time and resume it in another session.

### Subjects

Five subjects took part in the experiment, including one of the authors (G. W.). The other four subjects were paid students with no experience with psychophysical tasks. All subjects had normal or corrected to normal visual acuity. Only one of the subjects also participated in the preexperiment (Appendix C). However, we did not use his data from that preexperiment. Instead, this subject produced entirely new data in the main experiment.

This work was carried out in accordance with the Code of Ethics of the World Medical Association (Declaration of Helsinki) and informed consent was obtained for experimentation with human subjects.

### Results

**Figure 6.**
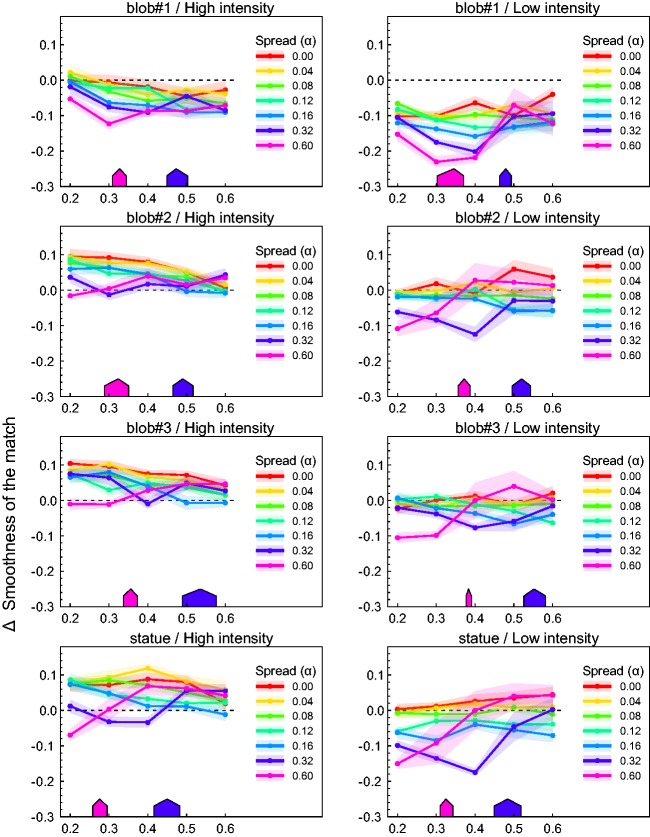

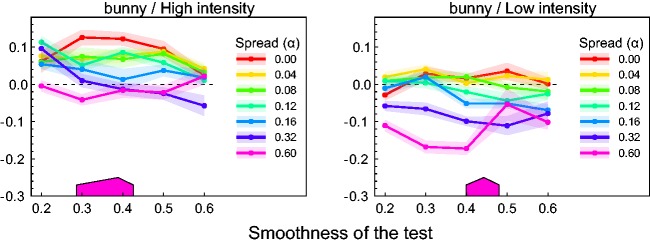
Results of Experiment 1, averaged across all five subjects. The diagrams differ with respect to shape condition (rows) and light intensity condition (columns). The original smoothness settings were transformed into the measure Δ smoothness. The mean Δ smoothness values for the different light spread conditions are shown in different colors. Transparent areas represent ±*SEM*. The colored roof-like symbols in each diagram are derived from our second experiment and indicate those positions on the smoothness axis where the highlight groups on the surface of the objects started to be perceived as sets of isolated highlights (see Figure 11 for details).

Figure 6 shows the results of the experiment, averaged over all five subjects. Each diagram presents the data for one of the five different shape conditions (rows) under one of the two light intensity levels (columns). To focus on the effects of the independent variables, we transformed the original smoothness settings into the difference measure “Δ smoothness” by subtracting the true smoothness value of the test stimulus. The settings made in the seven light spread conditions are shown separately in different colors.

In case of complete constancy, that is, if the glossiness perception of our subjects were completely unaffected by our experimental conditions, their settings would all be located on the dotted baseline in the plots. This is clearly not the case. We calculated a four-way analysis of variance (ANOVA) with the factors shape, scaled smoothness, light spread, and light intensity of the test stimuli, which resulted in highly significant main effects for all four factors as well as highly significant first-order interactions for all but the combination between shape and light intensity (for details see Appendix D).

**Figure 7.**
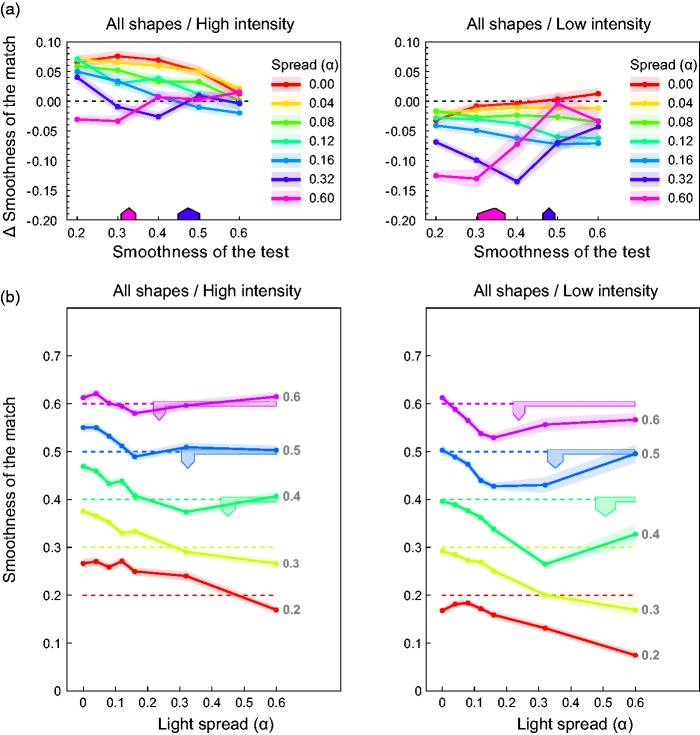
The glossiness settings of Experiment 1 averaged across subjects and shapes. (a) The settings plotted against the smoothness of the test surface with spread as grouping variable. The data correspond to an average across shapes of the data shown in Figure 6. (b) The same settings as in (a) but plotted against the light spread variable with smoothness as grouping variable (with the respective test smoothness value attached to the end of each curve). In all diagrams, the roof-shaped markers correspond to the range, where according to Experiment 2 overlapped highlight groups start to split up into distinguishable singular highlights. The transparent areas around each data curve represent ±*SEM*.

A pairwise comparison of the diagrams in each row of Figure 6 reveals that in general the perceived glossiness of the surfaces was considerably higher in the high-intensity condition (left column) than in the low-intensity condition (right column). The shape of the test object had also an influence on perceived glossiness. If one compares the diagrams within each column, it seems that especially blob#1 differs from all other shape conditions in that its glossiness was consistently underestimated under almost all conditions. However, the general pattern of the results was similar enough to justify the aggregation of the data across shapes. Figure 7(a) shows the corresponding mean results. Figure 7(b) provides a different view on the same data. Here, the glossiness settings are plotted against light spread α with the smoothness of the test as a grouping variable.

A closer look at the data curves depicted in Figure 7(a) reveals a seemingly complex interaction between the light spread variable and the smoothness of the test surfaces. At the lowest gloss level (smoothness value = 0.2), a simple and consistent pattern resulted: Objects with this low amount of surface gloss lose the more in perceived glossiness the larger the light spread and thus the difference between the illumination directions is. With increasing smoothness of the test objects, more differentiated effects are observed that also depend on the intensity level of the light sources.

Despite considerable noise in the data, the curves exhibit a characteristic pattern that is especially evident under the low-intensity condition (right column in Figure 7(a)): For relatively low light spread values (0.0–0.16), the order in perceived glossiness observed in the low smoothness level seems to be preserved at higher smoothness levels, that is, the single curves keep an almost constant distance to the zero reference line of perfect constancy. In the two highest light spread levels, however, the effect strongly depends on the smoothness level and a sharp and distinctive increase in glossiness occurs at certain positions along the curve. For the highest light spread of α = 0.6 (pink lines in the diagrams), such a disproportionate rise happens either between the second and the third smoothness level of the test (i.e., between a smoothness value of 0.3 and 0.4), or between the third and the forth one (i.e., between the smoothness values of 0.4 and 0.5), depending on the shape of the object. Under the second highest light spread (dark blue lines), this effect consistently occurs at a smoothness value that is at least one level higher. In most of the shape conditions, the curve for the highest light spread level flattens out immediately after that peak and reaches the level of the 0 spread condition (red lines) at the highest smoothness value.

The pattern in Figure 7(b) appears even more regular. The settings for each of the five test smoothness values show a characteristic pattern with increasing light spread. All curves first decrease monotonically. In the upper three curves with smoothness values >0.3, a minimum is reached at some spread level, after which the curve increases monotonically. With decreasing test smoothness, the location of this minimum shifts to higher and higher spread levels. This suggests that the minima in the lower two curves at smoothness levels 0.2 and 0.3 cannot be observed, simply because their locations fall outside the light spread range realized in the experiment.

Although qualitatively similar, these effects are less pronounced under the high-intensity condition (left columns in Figures 6 and 7). Relative to the low-intensity condition, the glossiness settings in the high-intensity condition are shifted to higher values and the range of the settings is somewhat lower. These differences can best be seen in Figure 7(b).

### Discussion

Our first explorations with a cylindrical object indicated that the perceived glossiness of simple surfaces may not only be influenced by the number but also by the relative positions of point light sources. These results further suggested that this effect also depends on the relationship between the direction of highlight variability and the local curvature of the surface (see Figure 3) and it was thus not clear, whether similar effects can also be observed in more complex surfaces that vary in curvature.

We explored this question with five shapes of different complexity. Our results confirm that clear effects of relative light point position on perceived glossiness can also be observed with more complex shapes. A second important observation is an interaction between light source spread and the objective smoothness level of the test surfaces: At low smoothness values, the perceived glossiness decreased systematically with increasing light spread, whereas the data indicate a clearly different effect for the two highest light spreads (α = 0.32, 0.6). In the latter case, a rather abrupt change in perceived glossiness occurred at certain smoothness levels (see the dark blue and pink lines in the diagrams in Figures 6 and 7(a)). This pattern is especially pronounced in the low-intensity condition.

**Figure 8.**
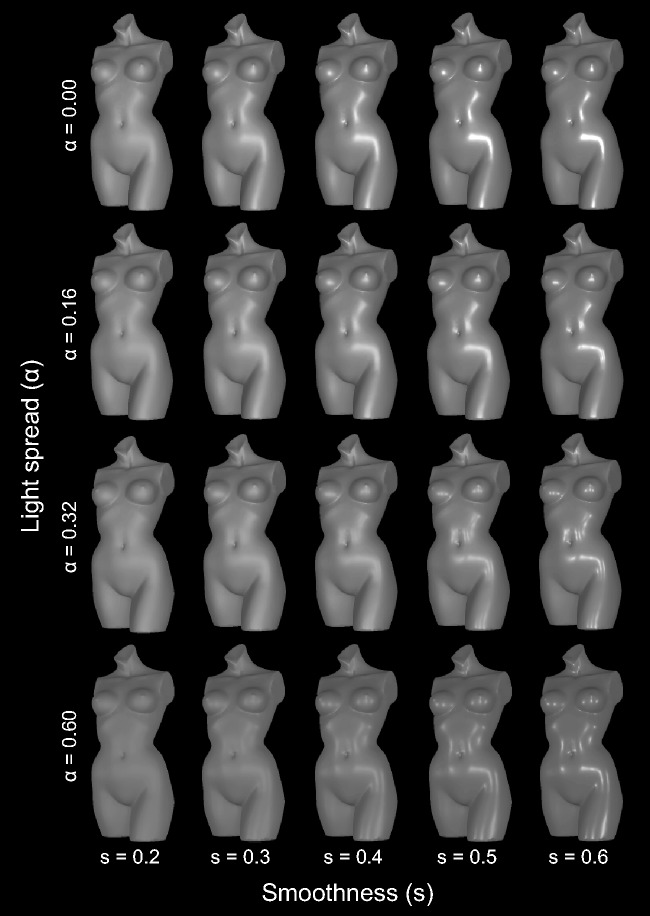
Shape condition “statue” for all gloss levels (columns) under four different light spread values (α = 0.0, 0.16, 0.32, and 0.6 from top to bottom). All stimuli were taken from the high-intensity condition.

An inspection of Figure 8, which shows the statue object under four different light spread values and all smoothness levels, helps to gain a more intuitive grasp of the underlying causes for this interaction between smoothness and light spread. At the lowest smoothness value of 0.2 (leftmost column), the highlights are relatively large, blurry, and of low contrasts and this leads to a rather matte appearance. Increasing the light spread at this level makes the intensity distribution more homogeneous and the surface appears even less glossy. This diminishing effect of light spread on perceived glossiness persists for the lower smoothness levels (≤0.3) over the whole light spread range: The higher the light spread, the lower the perceived glossiness of the surface. Accordingly, the data curves for these smoothness levels in Figure 7(b) decrease monotonically with increasing spread.

A comparison of the left and the right column in Figure 8 reveals that while the increase in light spread from top to bottom weakens the glossiness considerably for low smoothness values (left side), this is not the case for high smoothness values (right side): Although there is also a kind of change in the perceived material quality from top to bottom that is hard to describe, the glossiness as such seems much less affected by the same change in the light spread. This observation is reflected in the increase of the pink curve in Figure 7(a) from a negative glossiness effect at low smoothness values to a nearly zero effect at the highest smoothness value. Furthermore, the position of the disproportionate rise of this curve seems to occur at a smoothness value at which the overlapping highlights start to split up into separate highlights that are clearly discernible from each other. As a consequence of this split-up, the number of highlights increases while their width decreases. Both of these changes may contribute to an increase in perceived glossiness. In Experiment 2, we investigated and, to anticipate, found support for the hypothesis that these abrupt increases in perceived glossiness in our data are connected to a split-up of previously overlapped highlights.

This observation supports the assumption that local highlight features play an important role in gloss perception. One promising candidate for a local highlight cue for perceived glossiness is the blurring or the sharpness of the highlights, which is often related to the highlight width. Marlow and Anderson (2013) found in a recent study that the perceived sharpness of highlights could account for 96% of the variance of the glossiness judgments of their subjects that were obtained with test shapes similar to ours. Assuming that the perceived glossiness of our stimuli was mainly determined by the presence of this cue, it could actually explain most of our results, as we discuss in more detail later.

### The Effects of Contrast, Light Intensity, and Shape

Our data reveal additional effects of contrast, absolute intensity, and shape on perceived glossiness that seem in line with previous research.

Perceived glossiness was generally stronger under the high-intensity condition than in the low-intensity condition (left vs. right column in Figures 6 and 7). This may—at least in part—be attributed to an enhanced luminance contrast in high-intensity stimuli. It has repeatedly been found that the intensity contrast between the highlights and the diffuse parts of a surface plays a crucial role in glossiness estimation (Ferwerda, Pellacini, & Greenberg, 2001; Marlow & Anderson, 2013; Marlow, Kim, & Anderson, 2012). Especially for surfaces with a relatively low amount of gloss, this contrast information seems to be the dominant cue for glossiness (see Hunter’s “contrast gloss” category, 1937, 1975). An exact comparison of contrast levels is difficult, because it is at present unclear how a single contrast level can be assigned to stimuli containing a complex pattern of multiple highlights (Haun & Peli, 2013; Peli, 1990). For the sake of simplicity, we calculated three different contrast measures for each stimulus, namely, a simple Michelson contrast, a space-averaged Michelson contrast, and a space-averaged Whittle contrast (see Moulden, Kingdom, & Gatley, 1990), taking always only those pixels into account that belonged to the test surface, while ignoring the background color. Each of these contrast measures indicated an increase in luminance contrast in high-intensity stimuli. It should be noted that this increase is largely due to the ambient light component that adds a constant amount of luminance to each surface. Without this ambient component, the Michelson contrasts and other relative contrast measures (see Hunter, 1937) would have been invariant under changes in the intensity of the light source.

We assume that not only enhanced luminance contrast but also an increase in the absolute luminance level contributed to an increase in perceived glossiness. Although we are not aware of a study that explicitly investigated how gloss perception is affected by differences in the intensity of the illumination, there is indirect evidence that such an effect exists. Motoyoshi and Matoba (2012) varied a number of image statistics of an illumination map and tested how these manipulations affected the material impression of objects rendered with these illuminations. One of their results was that perceived glossiness increased with mean luminance of the illumination maps. There are additional studies which indicate that perceived glossiness also depends on the absolute intensity level of the highlights. Qi et al. (2014), for instance, used a measure representing the “highlight strength” of their computer-generated stimuli which was defined as the mean intensity of the highlights. They found a significant correlation of ρ = .77 between the highlight strength and the glossiness judgments of their subjects. In another experiment, Ferwerda and Phillips (2010) found that reducing the dynamic range of their stimuli also reduced the degree of perceived glossiness. These findings of Ferwerda and Phillips could also explain another aspect of our data: At a spread of 0, the data curves in the low-intensity condition are almost flat, whereas they decline with increasing smoothness in the high-intensity condition (compare the red lines between the two diagrams in each row in Figure 6). It is conceivable that this discrepancy is due to the limited intensity range of our stimuli. We did not use tone mapping to rescale the dynamic range. Pixel values exceeding the maximum intensity were simply clipped which was more likely to happen for stimuli with higher smoothness parameter values under the high-intensity condition. In fact, we found that 38 of our 350 stimuli contained pixels at the upper intensity limit (with rgb = 1.0, 1.0, 1.0) that all belonged to the high-intensity condition under smoothness values of 0.5 and 0.6 and almost exclusively occurred under light spread values between 0.0 and 0.12 (except for three conditions with less than 5 pixels exceeding the maximum intensity level which occurred under a light spread value of 0.16). It is reasonable to assume that the surfaces would have been judged as even glossier and that no decline in the curve would have occurred, had it been possible to display the entire intensity range of such overshooting highlights. However, since clipping was restricted to a few specific cases in the high-intensity condition, this potential problem does not seriously limit the interpretability of our results.

Our data also indicate an influence of shape on perceived glossiness. Most salient is the difference in perceived glossiness between blob#1 and the rest of the shapes. In blob#1, glossiness was consistently underestimated under almost all conditions. Object blob#1 was closest to the shape of a sphere and thus had lower local curvatures than the other shapes. As Figure 2 demonstrates, lowering the curvature leads to broader and more blurry highlights and as a consequence to a reduction in perceived glossiness (see also Nishida & Shinya, 1998; Wendt et al., 2010). Together, this seems sufficient to explain the observed effects of shape.

## Experiment 2

**Figure 9.**
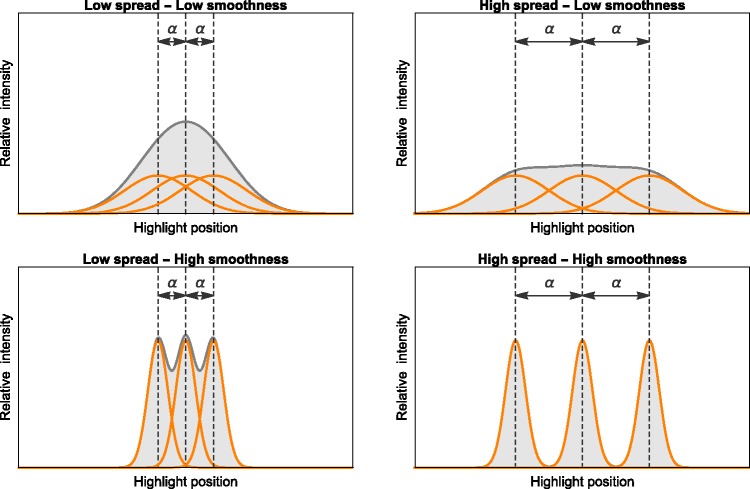
Superposition of highlights belonging to different light sources. The width and intensity of the merged highlight depend on both surface smoothness (top vs. bottom) and light source spread α (left vs. right). The graphics demonstrate the interaction between spread and smoothness: An increase of the spread in the depicted range widens the single highlight on a surface of low smoothness (top row). On a high smoothness surface the same increase in spread leads to a split-up into individual highlights with much smaller widths (bottom row). As a consequence, perceived glossiness decreases with increasing spread for low smoothness and increases for high smoothness.

The most prominent finding of Experiment 1 was that perceived glossiness increased sharply under certain stimulus conditions. Informal observations suggested that this effect was related to a split-up of a single merged highlight into its superpositioned constituents that individually had a much smaller width. The fact that steep increases in perceived glossiness only occurred in the two largest light spread conditions supports this explanation, because the split-up of overlapped highlights should for very smooth surfaces occur at a lower spread value than for less smooth surfaces (see Figure 9).

If this explanation is correct, then the rise in perceived glossiness should coincide with the perceptual split-up of the merged highlights. It is obvious that a test of this prediction requires that one knows the degree of overlap at which the visual system considers three superpositioned intensity distributions as separate. In our informal inspection of the example stimuli in Figure 8, we used the existence of a distinct gap between adjacent highlights as a pragmatic criterion. However, the detection threshold of the visual system could be considerably lower—especially with our stereoscopic set up, since the availability of highlight disparity information in our stimuli could potentially support the segmentation process (Wendt et al., 2008). We therefore conducted a second experiment to determine the parameter values at which this transition in the highlight patterns occurred. The results were then related to the results of our first experiment.

### Stimuli and Procedure

The general set up of the scenes was the same as in Experiment 1: We used the same five shapes (Figure 5) which were rendered under the same five different smoothness values (from 0.2 to 0.6 in steps of 0.1). We again used three point light sources, each with a constant distance of 5 units to the center of the test object (see Figure 4). However, the light spread was now a dependent variable and the subjects’ task was to find the minimum value of the spread, at which overlapping highlights become discernible according to a given criterion. As one of the reviewers of an earlier draft of this article mentioned, this task has a certain resemblance to procedures used in optics to determine the resolving power of an imaging system (see, for instance, the “Rayleigh criterion”; Rayleigh, 1879).

To determine an upper bound for detection performance, we used three point lights with different colors in some conditions, because this should facilitate the distinction between them. In these cases, the center light (see Figure 4) was red (rgb = 1.0, 0.0, 0.0), the left light green (rgb = 0.0, 1.0, 0.0), and the right light blue (rgb = 0.0, 0.0, 1.0).

All three lights had the same intensity of 1.5. For a light spread of 0, the superimposed colored lights were spatially in register and their additive mixture appeared white.

With increasing light spread, the colors of the diverging highlights become more and more saturated. These chromatic transitions were used in the first three detection criteria:

1. “Gap”: Adjust the minimal light spread such that a just noticeable gap between all three colored highlights of a group can be seen.
2. “All colors”: Adjust the light spread such that the three different colors are just distinguishable from each other. Compared to the first task, a certain degree of overlap between the highlights is to be expected under this instruction.
3. “No red”: Adjust the minimal light spread such that the red color of the center highlight is not yet detectable, while at the same time the colors of the two flanking green and blue highlights can be distinguished. We expected the highest degree of overlap under this condition.
4. “White high,” “White low.” Here the original conditions of the first experiment were used, that is, all point lights were white and their intensity was either 0.5 (“low”) or 1.5 (“high”). The subjects were asked to adjust the light spread such that the highlight groups just start to appear as a set of individual highlights, without stipulating any specific criterion.

Due to different local curvatures within a surface, it is well possible that the spread values needed to fulfill the aforementioned criteria could vary across several positions of the surface. Therefore, the subjects were instructed to always refer to that highlight group where the criterion is met first (i.e., to use the smallest light spread value under which this criterion is reached).

In each trial, the light spread parameter started at a value of 0. Using the left and right arrow keys of the keyboard, the subject adjusted this parameter in a range between 0 and 1 until the respective criterion was met. In case the subjects reached the upper limit, the message “Maximum reached!” appeared in red letters on the screen underneath the test object.

It was possible, especially for low gloss stimuli with large and blurry highlights, that a criterion could not be fulfilled, because even the largest setting for the light spread was not sufficient to separate the highlights enough from each other to make them distinguishable. We therefore asked the subjects after each setting to indicate whether it was possible to meet the criterion by selecting between “Is feasible!” and “Is NOT feasible!.” During the adjustment, a short text described the relevant criterion in a few words (like “There is a just noticeable gap between the three colors” or “All three colors are just distinguishable from each other”). Although the four different instructions were used in separate blocks, we wanted to make sure that our subjects were always aware about the current criterion, since generally they completed several blocks in a row.

Each stimulus combination was presented 4 times during a block, so for the criteria 1 to 3 each block contained 100 trials (5 Shape Conditions × 5 Smoothness Levels × 4 Repetitions), and for the fourth criterion, the block contained 200 trials (5 Shapes × 5 Smoothness Levels × 2 Intensity Levels × 4 Repetitions). Within each block, the stimuli were presented in random order and the subjects had as much time as they needed to complete a session.

### Subjects

Four subjects participated in our second experiment. All had normal or corrected to normal visual acuity and normal color vision, as tested by means of Ishihara plates (Ishihara, 1967). One of the subjects was an author of this study (G. W.).

### Results

We first excluded all settings that were marked as “not feasible.” Three of the 2,000 settings were afterwards changed from “feasible” to “not feasible” to correct misclassifications that were reported by two of the subjects. In conditions, in which none of the settings was feasible, the light spread value was set to the maximum value of 1.

**Figure 10.**
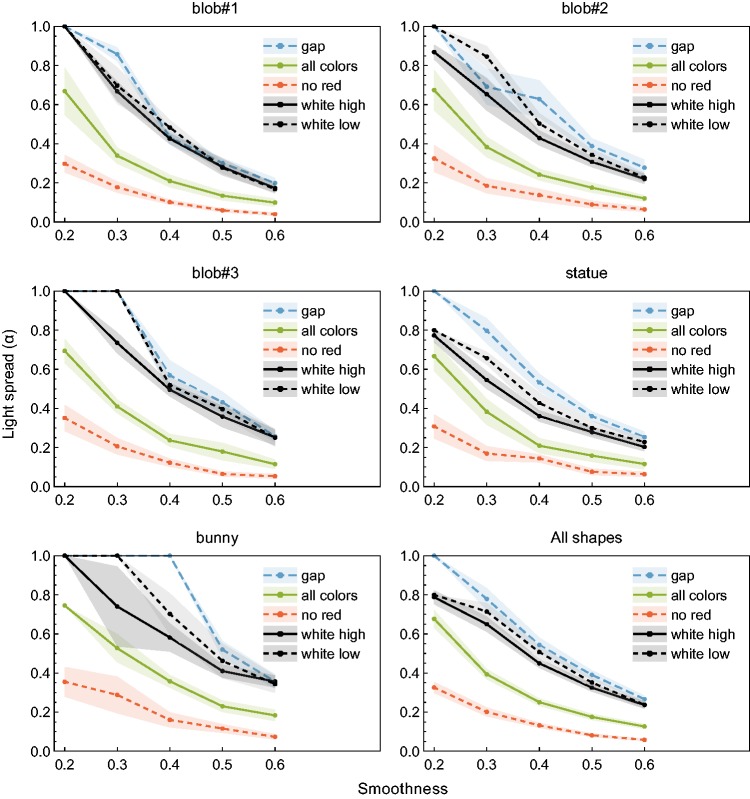
Light spread settings for different overlap criteria measured in Experiment 2. Each diagram shows the results for one of the five shapes used in both experiments. The data points are light spread settings averaged across all four subjects. Only settings that were marked as “feasible” by the subjects were included. The transparent areas around each curve represent ±*SEM*.

Figure 10 summarizes the remaining data averaged across all four subjects. The curves in each diagram connect the data obtained for one of the five criteria. The curves are all similar in shape and decrease monotonically with increasing smoothness. The subjects needed comparatively high values of the light spread in the “gap” condition (dashed blue lines in the diagrams of Figure 10). For low smoothness levels, it was often impossible to find a light spread that was high enough to meet this criterion. The severity of this restriction depended also on shape and was most pronounced for the bunny object. The settings for the “all colors visible” condition were not affected by this limitation, because here much lower spread values were sufficient to meet the criterion (solid green lines in Figure 10). As expected, the lowest light spread values resulted for the “no red” condition (dotted red lines in Figure 10). However, almost all of our subjects had difficulties with this task so that a large part of the trials across all smoothness levels were marked as “not feasible.” According to the subjects’ reports, those parts of the highlight pattern that were mainly produced by the blue point light always also had a reddish tint to some degree when it was mixed with the neighboring red light. It was therefore difficult to decide, whether the criterion was fulfilled or not. The light spread settings for the two conditions with white lights (see Experiment 1) tended to be located between those made in the “gap” and the “all colors visible” condition, with a consistently lower detection threshold in the high-intensity (solid black lines) than in low-intensity (dashed black lines) condition.

The main purpose of our second experiment was to determine the smoothness values, for which the overlapped highlights caused by different light sources just start to appear as separated. This was motivated by the assumption that the disproportionate increase of perceived glossiness observed at some smoothness values in Experiment 1 (see Figure 7(a)) was due to such a split-up of overlapped highlights into much smaller separate ones. If this hypothesis is correct, then the position of the split-up should coincide with the position of the abrupt increase in perceived glossiness.

**Figure 11.**
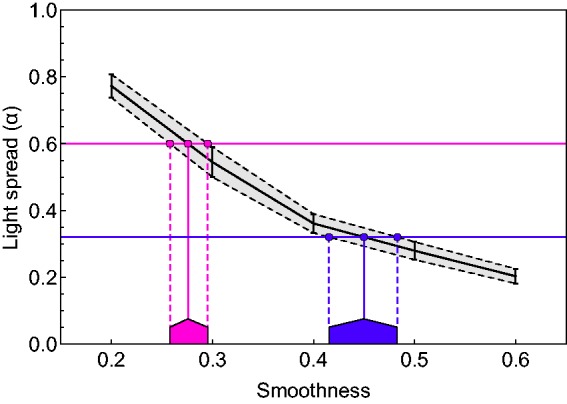
The method used to determine the smoothness value of a surface at which a highlight group starts to appear as a set of isolated highlights for a certain light spread level, illustrated for the statue object under the high-intensity condition. This point, that is represented by the peak of the roof-shaped markers, was determined by projecting the intersection of a horizontal line through the relevant light spread value (here, 0.32 and 0.6) with the data curve from Experiment 2 (black solid line). The left and right endpoints of each marker are determined by the intersections between the horizontal line and the mean ± 2 *SEM* curves (dotted lines).

Using the data curves under the “white low” and “white high” conditions, we each determined the respective locations for the two highest light spread levels 0.32 and 0.6, for which such abrupt changes were observed in Experiment 1. Figure 11 illustrates how this was done for the statue object under the high-intensity condition (compare the solid black line in the corresponding diagram in Figure 10): To find for both light spreads (0.32 and 0.6), the minimum smoothness values at which separate highlights can be seen, we determined the *x*-coordinate of the point where the respective curve intersects a horizontal line through the ordinate values 0.32 and 0.6, respectively. In Figure 11, this point is indicated on the smoothness axis by dark blue and pink, respectively, roof-like markers, whose peaks refer to the intersection with the mean curve and the flanks to the positions of the intersection with the curves through mean ± 2 *SEM*. These markers are added to the corresponding diagrams in Figures 6 and 7(a), to allow a direct check of how well the data of our second experiment predict the abrupt changes in the data curves of Experiment 1.

In the alternative representation of our data in Figure 7(b), the minimum in the glossiness settings should occur at a light spread that is just large enough to make individual highlights discernible. To also allow a direct test of this prediction, we used the data of Experiment 2 to indicate this critical light spread in Figure 7(b). This is simply the light spread setting made in the “white” condition for the corresponding smoothness level.

### Discussion

The fact that the curves for the two white light conditions are located between those obtained for the “gap” and the “all colors visible” condition indicates that the visual system classifies highlights as separate even if their spatial intensity distributions overlap to some degree.

Under the “white high” condition, there was the potential problem that the subjects could produce highlight patterns whose intensity peaks exceed the maximum intensity level due to large overlaps between the highlights of a group. In such cases, the intensity was simply clipped at the maximum intensity level and this could have resulted in an unnatural appearance of affected highlights. However, it is highly improbable that such distorted highlights actually occurred, because—as the analysis reported in the discussion of Experiment 1 has revealed—such cases are almost exclusively found for light spread values less or equal to α = 0.12, whereas the α-settings under the “white high” condition in the present Experiment were generally well above 0.12 (with a minimum setting at α = 0.125).

A check of the marker positions in the diagrams of Figures 6 and 7(a) reveals that most of them are close to the smoothness range at which the steep increase in the glossiness settings took place (with the notable exception of the blob#1 object, where a significant deviation occurs; see the pink symbol in the top right diagram in Figure 6). In Figure 7(b), the markers are close to the spread levels at which the minimum in the glossiness settings occurred.

The results from our second experiment also predict those two cases under the bunny shape condition where an increase in perceived glossiness is not observed: Since the respective data curves of Experiment 2 did not intersect the light spread scale at a value of 0.32 (i.e., the dashed as well as the solid black curve in the bunny diagram in Figure 10), no dark blue markers could be added to the respective diagrams in Figure 6. The results also indicate that the minimum spread level at which individual highlights can be distinguished for stimuli with smoothness values ≤0.3 is larger than the spread realized in Experiment 1. This explains why in Figure 7(b) a minimum is missing in the two conditions with the lowest smoothness values.

All in all, the present results support our assumption that the disproportionate rise in perceived glossiness took place at a point in parameter space at which the highlight groups on the surface perceptually started to split up into sets of isolated highlights.

## Test of Global Glossiness Cues

Our experimental manipulations influence the highlight pattern in the proximal stimulus in predictable ways. The central observation is that independent highlights belonging to different light sources overlap in the stimulus to some degree and thus form highlight groups. The spatial relation of the highlights depends mainly on the relative positions of the light sources and the degree of overlap is influenced by both surface smoothness and the distance between the lights. A natural question is, whether the causal relationships between these factors that influence the specific structure of the highlight pattern are taken into account by the visual system when estimating the glossiness of a surface.

This question is of interest, because there is some evidence indicating that the visual system relies on several global image cues to estimate the material properties of an object. Marlow and colleagues (Marlow & Anderson, 2013; Marlow et al., 2012) found that perceived gloss could be well predicted by a linear combination of three different image features: (a) The intensity contrast between the highlights and the diffuse parts of the surface, (b) the sharpness of the mirror images of the environment, and (c) the coverage, that is, the relative proportion of the surface that is covered with specular reflections. Using surfaces with complex mesoscale structures as stimuli, Qi et al. (2014, 2015) examined further image features, such as the number of highlights, their size, strength (i.e., the mean intensity of the highlights) and spatial distribution as well as the percentage of highlight area, that is the relative proportion of the surface that is occupied by highlights. They found significant correlations between most of these image cues and glossiness judgments and also suggested a linear model to predict perceived glossiness. Qi et al. (2014, 2015) varied the mesoscale geometry of their surfaces while keeping the illumination constant, whereas our focus was on varying the illumination. Nevertheless, the image cues they proposed seem well suited to also analyze the present results, because they are systematically affected by the factors varied in our experiment: In general, increasing the light spread reduces the strength of the highlights and at the same time increases the percentage of highlight area. Other image features are influenced by the smoothness of the surface. To test whether the glossiness judgments made in Experiment 1 can also be predicted by a linear combination of the proposed image statistics, we evaluated each of our stimuli using the same procedures as Qi et al. (2015), which are based on image processing techniques. As will be shown later, this was actually not very successful. In a first attempt to improve on these results, we focused on the method used to segregate the gloss layer from the stimulus, that is, to determine the stimulus regions that are covered with highlights. Qi et al. (2015) used a simple fixed decision rule to determine whether or not a pixel belongs to the gloss layer (see next section). As an alternative, we employed a segmentation method that was based on empirical data from a matching experiment. In this experiment, the perceived highlight extension of each test stimulus was matched in a two-colored comparison stimulus (Appendix E).

### Methods

Qi et al. (2014, 2015) proposed five different image statistics. We considered only the four most predictive ones, namely, the strength, number and mean size of the highlights, as well as the percentage highlight area.

**Figure 12.**
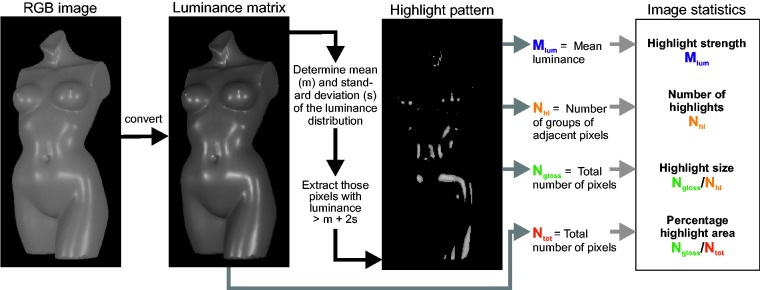
Illustration of the procedure used to extract the four global image statistics proposed by Qi et al. (2015).

Following the procedure described in Qi et al. (2015), we converted each gray-scale image of our test stimuli into a matrix of luminance values (see Figure 12). The matrices contained both half-images of the stimulus side by side, just as they appeared on the monitor screen during Experiment 1. The background color was ignored in all calculations. All pixels with a luminance larger than mean +2 *SD* of the images’ luminance distribution were considered to belong to the highlight area. For the resulting highlight images, the following single image features were calculated: The number of the highlights was defined as the number of connected areas in the highlight images (using a connectivity of eight neighboring pixels) divided by two, since we took both half-images into account. The mean size of the highlights was the number of pixels within the highlight images divided by the total number of connected highlight areas (i.e., for both half-images), the percentage highlight area was the number of highlight pixels divided by the number of pixels of the entire stimulus (without the background) and the strength was the mean luminance of the highlight pixels.

### Results

The four image statistics were used to predict the mean smoothness judgments made for the 350 different stimuli used in Experiment 1. Across the two methods that we used to determine the gloss layer of a stimulus, 16 stimuli for which no highlight area could be extracted were marked as “invalid cases.” This was either because none of their pixels met the corresponding criterion or because their highlight extensions were set to 0 in our empirical experiment (Appendix E). We also added those 38 stimuli to the group of invalid cases that contained pixels at the upper intensity limit (at rgb = 1.0, 1.0, 1.0), since their highlights bore the risk of having a distorted appearance due to clipping (see the Discussion section of Experiment 1). Thus, in total, 54 invalid cases were excluded from further analysis.

**Figure 13.**
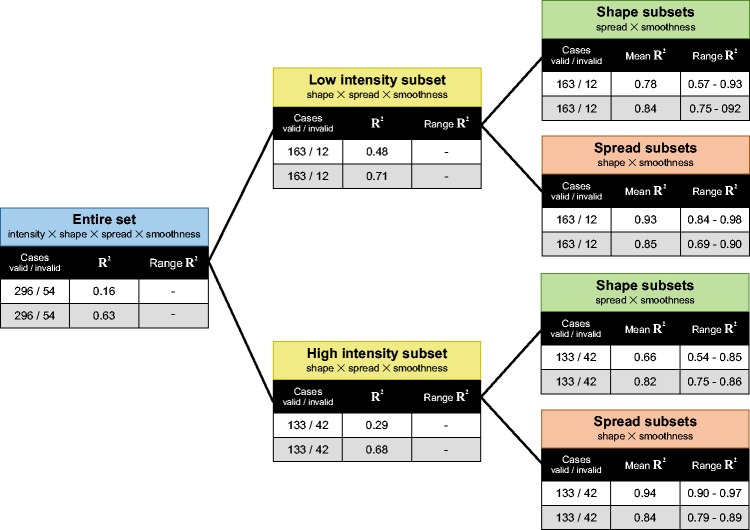
The predictability of the perceived glossiness of our stimuli by a linear combination of four different image statistics is shown for different combinations of the factors light intensity, shape, light spread, and smoothness of the surface. The first data row in each block (white cells) shows the results that were obtained using the gloss layer separation method provided in Qi et al. (2015), the second row (light gray cells) those for our empirical method (see Appendix E). With both methods the proportion of explained variance *R*^2^ increases when the number of factors to be combined is reduced (from left to right). However, especially for combinations of more than two factors (blue and yellow blocks) the *R*^2^ differ significantly between the two methods, suggesting that the algorithm used by Qi et al. (2015) to segregate the gloss layer is inappropriate. Of our different 350 stimuli, 54 were excluded from the calculations (which we refer to as invalid cases; see the left column within each block) either because no highlight area could be extracted from these stimuli or because they contained highlights that were clipped at the upper pixel intensity limit.

In a first step, the four image statistics were separately correlated with the smoothness settings. For those image statistics that were calculated using the segmentation method by Qi et al. (2015) (white cells in Figure 13), we found a weak negative correlation between the size of the highlights (ρ = −.28) and perceived glossiness, while the other statistics showed even lower correlations (highlight strength: ρ = −.19, number of highlights: ρ = .19, and percentage highlight area: ρ = .005). Even the combination of the four image statistics in a multiple linear regression model could only account for about 16% of the variance of the smoothness settings—which is considerably less than the proportion of explained variance of 97% reported by Qi et al. (2015). In scatterplots relating the mean smoothness settings to the single image statistics, we found pairs of clusters in some cases that turned out to be associated with the two different intensity levels realized in Experiment 1. We therefore applied a separate multiple linear regression model for both intensity levels and found a moderate enhancement of the explained variance (*R*^2 ^= 0.48 for the low-intensity stimuli and *R*^2 ^= 0.29 for the high-intensity stimuli; see the yellow blocks at the second level in Figure 13).

Splitting up our data set by yet another factor so that only combinations of light spread and smoothness were checked separately for all shape conditions (green data blocks in the third level in Figure 13) led to a further increase in the proportions of explained variance, but it was still lower than in the study of Qi et al. (2015): In the low-intensity condition, the *R*^2^ values for the five different shape conditions ranged between 0.57 and 0.93 (mean *R*^2 ^= 0.78) and in the high-intensity condition between 0.54 and 0.85 (mean *R*^2 ^= 0.66).

Considerably higher *R*^2^ values were obtained when combinations of shape and smoothness were tested separately for all intensity and light spread levels (see the orange blocks in Figure 13): For these subsets, the proportions of explained variance were comparable to those reported by Qi et al. (2014, 2015). The *R*^2^ values ranged between 0.84 and 0.98 for the low-intensity subset and between 0.9 and 0.97 for the high-intensity subset. In these cases, the highlight strength was the best predictor of perceived glossiness with correlation coefficients between 0.84 and 0.97.

In the results obtained with our empirical method to segregate the gloss layer from the stimuli (light gray cells in Figure 13), a different picture emerges: The combination of all four image statistics could explain more than 63% of the variance in the smoothness settings from Experiment 1 (compared to only 16% under the other method). Here, the strongest contributor was the image statistic “percentage highlight area” with ρ = −.69 (highlight size: ρ = −.41, highlight strength: ρ = .23, and number of highlights: ρ = .16). A split-up of the data set into the two intensity subsets led to only small enhancements of the *R*^2^ values, which were nevertheless considerably higher than those under the other segregation method (0.71 and 0.68 for the low and the high-intensity subsets, respectively, compared to 0.48 and 0.29). Again, even higher coefficients of determination were found for those subsets where only two of our experimental factors were varied: For the shape subsets (green blocks in Figure 13), where only the light spread and the smoothness were varied in the stimuli, we found an average proportion of explained variance of 84% for the low-intensity group and of 82% for the high-intensity group (compared to *R*^2 ^= 0.78 and *R*^2 ^= 0.66, respectively, that were obtained under the other method). Although, in general, the image statistic “percentage highlight area” was still the most predictive one for these subsets, there were at least two shape conditions (“blob#2” and “statue,” both under the low-intensity group) where the image statistic “highlight size” provided the strongest predictor for the perceived glossiness (with ρ = .82 and ρ = .85, respectively). The results for those subsets where only the factors shape and smoothness were varied are similar (orange blocks in Figure 13): On average, the four image statistics could explain 85% of the variance in the smoothness settings for the low-intensity group and 84% for the high-intensity group—which is actually less than the *R*^2^ values obtained under the alternative algorithmic segregation method (with average *R*^2^ values of .93 and .94, respectively). For the majority of these subsets, the strength of the highlights was the most predictive image statistic, followed by the “percentage highlight area.” However, in one case (stimuli with a light spread parameter value of 0.6 under the high-intensity condition), the image statistic “highlight size” contributed most to the explained variance (with ρ = .79).

### Discussion

Our analysis shows that the predictability of the perceived glossiness of our stimuli by a linear combination of four image statistics critically depends on the method used to extract the gloss layer from the stimuli. Generally, our empirical method (see Appendix E) leads to considerably better predictions compared to the strictly algorithmic method used by Qi et al. (2015).

**Figure 14.**
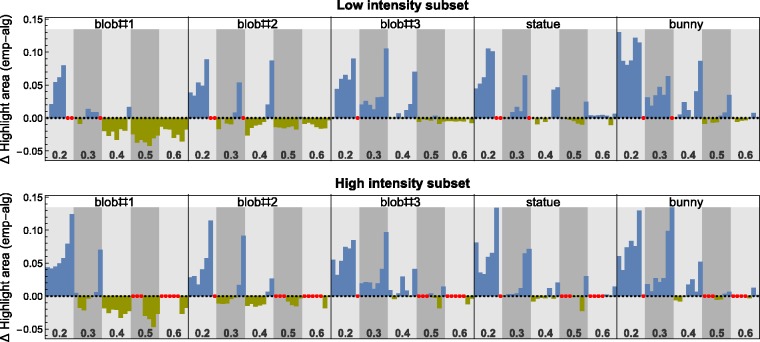
Direct comparison of the relative size of the extracted gloss layer between the two segregation methods. For each of our test stimuli (abscissa) we calculated the difference (Δ highlight area) of the relative size of the gloss layer (“percentage highlight area”) between the empirical method and the algorithmic method as proposed by Qi et al. (2015), that is, positive values (blue bars) represent larger highlight areas for the empirical method, negative values (green bars) larger areas for the algorithmic method. The stimulus conditions are sorted by the intensity level (low-intensity subset in the top panel, high-intensity subset in the bottom), the different shape conditions, the five test smoothness values (grayish segments within each shape block) and the 7 light spread values (dots within each smoothness segment, from left to right in ascending order). Stimuli that were marked as invalid cases were set to Δ highlight area = 0 (red dots).

If one compares the relative sizes of the extracted highlight areas for each of our stimuli between the two different methods, one can see that for higher smoothness levels, these sizes were similar (Figure 14). However, for lower smoothness levels, especially in combination with higher light spread values, the sizes of the algorithmically segmented highlight areas were systematically smaller than those obtained with our empirical method. Further analyses suggest that the algorithmic method did not only lead to an underestimation of the sizes of the single highlights, but that in many cases highlights are completely missed. We compared the image statistic “number of highlights” and found that in 78 of the 296 valid cases identical numbers of highlights were found by the two methods. In 74 cases, the algorithmic method detected more highlights, almost half of them (36 cases) under stimulus conditions where the shape “bunny” was used. Due to the specific mesoscale structure of this shape, the gloss layer was generally highly scattered (see Figure 5). In the remaining 144 cases (i.e., for 48% of the valid stimuli), the empirical method yielded more highlights. Under the assumption that the empirical method allows a more accurate identification of highlight structures, this would mean that in these cases the intensity profiles of at least some highlights were too low to be detected by the algorithmic procedure. In the current version of this method, the intensity threshold is based on the global luminance distribution of the stimulus and all pixels that exceed the mean luminance by two standard deviations or more are considered to belong to the gloss layer. The factor of two seems rather arbitrary and for an improved detection performance it might be necessary to adapt this factor to other, as yet unknown, stimulus features. It is even conceivable that the luminance threshold depends on local rather than global stimulus characteristics.

While the algorithmic gloss segmentation seems not optimal, our results suggest that a linear combination of the *image statistics* proposed by Qi et al. (2015) can be used to predict the perceived glossiness of our stimuli rather well. Although the predictive power of the linear model systematically decreases with the number of influencing factors (Figure 13), we obtained proportions of explained variance between 63% and 71% for those data sets, where three or even all four experimental factors were varied (blue and yellow blocks in Figure 13). For smaller subsets with two combined factors, proportions between 82% and 85% were found. Interestingly, there is one case where the algorithmic segmentation method leads to higher proportions of explained variance: For stimulus subsets where only the shape and the smoothness of the surfaces were varied (compare the results in the orange blocks in Figure 13), that is, those object properties that have also been investigated by Qi et al. (2014, 2015), we found *R*^2^ values that were comparable to those reported in Qi et al. (2015). It is not fully clear why the empirical method is inferior in this case. However, there are some indications that difficulties in applying the empirical method in one of our shape conditions, namely, “blob#1” (see Figure 5), is the main reason: (a) As can be seen in Figure 14, the segmented highlight area resulting from the empirical method is generally larger than that obtained with the algorithmic method. For the shape condition “blob#1,” however, this trend was reversed for the majority of the cases, especially under stimulus conditions with smoothness values larger than 0.3. (b) The finding that the rating of the match quality was significantly lower in “blob#1” than in all other shape conditions (see Appendix E) indicates that it was comparatively hard to achieve a satisfying match in this condition. (c) Finally, excluding data from “blob#1” actually leads to a moderate improvement in prediction quality under the empirical method for the “spread subsets” (see orange blocks in Figure 13): The mean coefficients of determination went up from 0.84 and 0.85 (for the low-intensity and the high-intensity subset, respectively) to 0.91 for both intensity subsets, while under the algorithmic segmentation method the respective values stayed nearly constant (0.95 for both intensity subsets). Although the proportions of explained variance are still higher under the algorithmic method, it seems that, at least in part, the difficulties of our empirical method with shape condition “blob#1” contributed to the comparatively weaker outcome in those stimulus conditions where only the factors shape and smoothness were varied.

In general, our results seem to support the idea that the visual system makes use of certain global image statistics to judge the material properties of surfaces. From the set proposed by Qi et al. (2014, 2015) especially the “percentage highlight area” and the strength of the highlights (or their mean intensity) seem to provide relevant information. However, contrary to Qi et al. (2014), who report a strong positive correlation of ρ = .90 between the image statistic “percentage highlight area” and perceived glossiness, we found a negative correlation of ρ = −.69 between these two measures. In order to find an explanation for this apparent discrepancy, it seems useful to identify stimulus variables in the two studies that contributed to the variability in “percentage highlight area.” With respect to Qi et al. (2014) this is obvious, because they used mesoscale surface roughness as the single independent variable. The positive correlation between “percentage highlight area” and perceived glossiness can be explained by the fact that both are in a very similar manner nonmonotonically related to the roughness variable. The main influencing factors in *our* experiment are the light spread and the microscale smoothness of the surface: While an increase in light spread generally leads to an increase in highlight area (see Figure 9), an increase in smoothness systematically reduces the size of single highlights and therefore also the total highlight area of the surface (see Figure 1). This means that light spread is positively correlated with “percentage highlight area,” whereas the microscale smoothness is negatively correlated with this image statistic. From a physical point of view, only microscale roughness is directly linked to the material properties of a surface (see Figure 1). Our finding that perceived surface glossiness is also negatively correlated with the “percentage highlight area” seems in agreement with this regularity. Roughly speaking, if the visual system uses the “percentage highlight area” as a cue for glossiness, it would use it “correctly” if it judges a surface the glossier the smaller the highlight area is.

The actual relationships are even more complicated, because they also depend on the kind of illumination in the scene as well as on the exact definition of “highlight area.”

Marlow and Anderson (2013) used a similar image feature, the “coverage,” which they define as the “proportion of a visible surface area that is occupied by specular reflections” (p. 1). Their definition is more general as the one used by Qi et al. (2014, 2015), who only considered highlights as part of the gloss layer and correspondingly used an intensity based criterion for their image statistic (namely, the relative number of pixels of a stimulus that exceed a certain luminance threshold, see Figure 12). In contrast, the coverage as defined in Marlow and Anderson (2013) includes any kind of features on a surface that appear as specular reflections of the environment. The relevant image information seems to be the presence of more or less sharp edges in the structure of the mirror images (Kim, Marlow, & Anderson, 2012; Kim, Tan, & Chowdhury, 2016). In Figure 1 in Marlow and Anderson (2013), the authors demonstrate how coverage depends on the microscale smoothness of the surface: The higher the smoothness, the more detailed and pronounced the structure of a real-world illumination map that can be discerned in the mirror image on the surface. With more diffusely reflecting surfaces, only features of the illumination that produce strong contrasts remain recognizable and as a consequence the total area of the surface that contains such features becomes smaller. Hence, the physically correct relationship between microscale smoothness and coverage would here be a positive correlation.

This shows that the inherent uncertainty of an image feature is not only due to the fact that it can be affected differently by different factors, such as the lighting conditions, object shape, and microscale smoothness, but that also the relationship between this image feature and perceived glossiness may vary: Under one condition surfaces are perceived the glossier the larger the areas of specular reflections are, while under other conditions they appear the glossier the smaller the areas. This indicates that the visual system cannot use such cues in a rigid manner, but that it has to adapt the magnitude and the sign of their weights to the specific stimulus conditions. This is also suggested by results from Marlow and Anderson (2013), who found that the weights of the three image cues “coverage,” “sharpness,” and “contrast” that served as predictors for perceived glossiness can vary considerably, depending on the illumination, the geometric structure of the surface, and the orientation of the surface towards the observer.

The set of image statistics that we use does not include a statistic related to the sharpness of the highlights, although there is some empirical evidence (e.g., Kim et al., 2012; Marlow & Anderson, 2013; Marlow et al., 2012) that perceived sharpness of the specular reflections on a glossy surface plays an important role in the perception of glossiness (see the “Distinctness-of-image-gloss” in Hunter’s classification; Hunter, 1937, 1975).

It is plausible that the manipulations in Experiment 1 also influenced highlight sharpness: Although there are often strong intercorrelations between different highlight features when only the surface smoothness is varied (with increasing smoothness the highlight becomes smaller, sharper, and more intensive; see Figure 1), their relationship becomes more complex when additional context factors come into play (see also Marlow & Anderson, 2013). For instance, as can be seen in Figure 9, the steepness of the intensity gradient at the border of a highlight group may depend in complex ways on the combination of surface smoothness and light spread.

Therefore, it is well possible that a measure that represents the perceived sharpness of a highlight would make an additional contribution to the predictability of the perceived glossiness of our stimuli. Extracting such a sharpness measure from a stimulus, however, would require more sophisticated image processing techniques. For the present set of image statistics, it was sufficient to count the pixels of a stimulus that met a certain intensity criterion or to average their luminance—and it was in part this simplicity that motivated us to apply this model.

Another problem with this model is that it has not yet been examined how strongly the single image statistics actually correlate with the corresponding perceived highlight features. Our finding that there are noticeable differences between the perceived extension of the highlight pattern and the image statistic “percentage highlight area” suggests that such problems actually exist and potentially have a strong impact on the predictive power of the image statistic. In addition, the usage of a simple intensity-based criterion can lead to errors in the detection of specular highlights if the 3D shape of the surface is not taken into account. As Marlow and colleagues have shown (Marlow & Anderson, 2015, 2016; Marlow, Todorović, & Anderson, 2015), the same luminance pattern can appear either as a matte or a glossy surface, depending on perceived local curvature. A mechanism that only relies on photometric information without putting them into a geometric context would be unable to detect such differences.

Marlow and Anderson (2013) avoided such problems by determining the perceived properties of specular reflections empirically: In a series of independent pair–comparison experiments with four different groups of subjects, they asked one group to judge the glossiness of a number of computer-generated 3D objects. These objects with highly specularly reflective surfaces were systematically varied with respect to their local curvature and were rendered under three different real-world illumination maps. The subjects in the remaining experimental groups had to judge the extent to which one of the three image cues “coverage,” “contrast,” and “sharpness” were present in the specular reflections of the same set of stimuli. Marlow and Anderson (2013) found that for such objects (that had similar mesoscale curvatures as ours), perceived sharpness was the strongest predictor for the perceived glossiness of the stimuli, which alone accounted for about 96% of the variance in the glossiness judgments.

## General Discussion

In the present study, we investigated how the perceived glossiness of a surface varies with systematic changes in the geometry of the light field and how potential effects of this manipulation depend on other context variables, such as object shape and the intensity of the light sources. As stimuli, we used computer-generated stereo images of complex 3D objects with a certain BRDF that were illuminated by three point light sources whose relative positions in space were gradually varied.

We found that each of the varied experimental factors had an influence on perceived glossiness: In agreement with previous findings (Nishida & Shinya, 1998; Vangorp et al., 2007; Wendt et al., 2010), our data show a systematic effect of object shape.

Surfaces with lower local curvatures (e.g., blob#1, see Figure 5) were judged as less glossy than those with higher local curvatures (e.g., bunny). The intensity of the light sources also affected perceived glossiness. In the high-intensity condition, the surfaces were perceived as considerably glossier than otherwise identical stimuli in the low-intensity condition. Our data suggest that both changes in the luminance contrast and in the absolute luminance level of the highlights contributed to this effect.

The novel aspect of our experiment is the smooth variation of the spread of three point light sources. These manipulations of the illumination had a clear and systematic effect on perceived glossiness and this effect was modulated by the smoothness of the surface. This shows that the illuminations that resulted from this manipulation of light source position are not functionally equivalent with respect to perceived glossiness. These findings complement results of other studies indicating that the appearance of a surface material also depends on the properties of the light field (Doerschner et al., 2010; Fleming et al., 2003; Motoyoshi & Matoba, 2012; Olkkonen & Brainard, 2010; Pont & te Pas, 2006).

The effects exhibited a characteristic pattern that was most pronounced in the low-intensity condition: For relatively small distances between the light sources (light spread α ≤ 0.16), the perceived glossiness decreased with increasing light spread. For higher light spreads (α > 0.16), however, an abrupt increase in perceived glossiness occurred at a certain smoothness level of the surfaces. We argued that this pattern can be explained by spatial properties of local highlight groups that are formed by the superposition of individual highlights that are related to different point lights. The degree of overlap between highlights in a group depends both on the smoothness of the surface (higher smoothness means smaller individual highlights and less overlap) and the angular distance between light source directions (larger angles lead to bigger shifts of individual highlights and thus less overlap). As long as the light spread is not large enough to make the individual highlights distinguishable, the group will be interpreted as a single highlight. An increase in light spread within this limit leads then to an apparent widening of the highlight and a corresponding decrease in perceived glossiness (see Figure 7). However, as soon as the light spread exceeds this limit, the highlight groups start to split up into several individual highlights that are much smaller and sharper leading to a sudden increase in the perceived glossiness of the surface. A further increase in spread has no additional effect. This is in line with this reasoning, because this influences only the spatial separation of individual highlights, but not their size. Additional evidence provide the results of Experiment 2, which show that separation of highlight and increase of perceived glossiness occur simultaneously.

At the highest smoothness level, the glossiness settings for a surface in the lowest spread condition were almost identical to those made in the highest light spread condition. This indicates that two lighting conditions that lead to clearly different highlight patterns can nevertheless have similar effects on perceived glossiness. This does not hold at lower smoothness levels, were these two light fields had considerably different effects on perceived glossiness. Thus, two different illuminations that have the same effect on perceived glossiness under a certain surface roughness will not necessarily produce same effects under other roughness levels. Our results indicate that the crucial condition for different light fields to have similar effects on glossiness is not that they produce similar highlight patterns on an object’s surface, but rather that the single highlights appear in an unbiased form. Their absolute intensity or the number of the highlights on the surface seems less important, because these features of the highlight patterns varied greatly between these two conditions.

One of the central observations of our experiment was that the glossiness of the surfaces is systematically underestimated when the highlights from different light sources appear as a single merged highlight. For instance, in the lowest smoothness level, the perceived glossiness of the surfaces decreased with increasing light spread. The explanation outlined earlier focuses on cues in the proximal stimulus and ascribes this effect to the flattening of the intensity profile at the location of each highlight group. From a more computational perspective, one may attribute this underestimation at least in part to the visual system’s inability to recover the actual cause for the change in the spatial intensity distribution. According to this view, the visual system erroneously interprets the changes in the proximal stimulus as a change in surface roughness, because the overlapping highlights provide not enough information to infer the true lighting conditions (see Pont & te Pas, 2006). When the highlight groups start to split up into individual highlights, however, the resulting, more complex highlight pattern may provide the visual system with sufficient information for a much better estimate of the true illumination conditions and this in turn increases the degree of gloss constancy. A number of studies indicating that under favorable conditions the visual system is able to construct an internal representation of the light field in the scene from shading patterns on objects (Kartashova, Sekulovski, de Ridder, te Pas, & Pont, 2016; Khang, Koenderink, & Kappers, 2006; Koenderink, Pont, van Doorn, & Todd, 2007) seems in line with this view.

In an additional analysis, we tried to predict the smoothness settings from a linear combination of global image statistics. To this end we adopted the model from Qi et al. (2015). The predictive power of this model in its original form was not very good. However, the predictions improved considerably after we replaced the algorithmic separation of the gloss regions in the stimulus used by Qi et al. by an empirical method. With this modification we found that especially the image statistics “percentage highlight area” (i.e., the proportion of a surface that is covered with highlights) and “strength of highlights” (i.e., their mean intensity) were strong predictors for perceived glossiness. Depending on how many influencing factors were varied in combination, we obtained proportions of explained variance between 63% (for the entire stimulus set where all four factors “smoothness,” “shape,” “light intensity,” and “light spread” were varied) and 85% (for subsets where only two factors were combined).

### Future Work

The results of our second experiment indicate that the visual system can make use of color information to disentangle the individual highlights of a group. Motion is another potential source of information that could facilitate the segregation process, especially when complex-shaped objects are considered. If objects like the ones used in the present study are rotated about an arbitrary axis, the highlights on its surface will successively pass through surface locations with different local curvatures, which in turn would cause changes in the spatial structure as well as in the relative distances between the single highlights of a group. We are currently investigating whether such color- and motion-induced information is taken into account and helps to improve gloss constancy in lighting situations that lead to merged highlights.

Furthermore, it seems worthwhile to extend the present investigation to more complex light source constellations. In our current experiments, the point lights were equally spaced along an arc (see Figure 4). The degree of overlap of independent highlights within different highlight groups across the surface was therefore similar. A more heterogeneous highlight pattern with different degrees of overlaps could be produced by independently varying the angles between the point lights in the scene. In extreme cases, both isolated highlights and groups of largely overlapping highlights could be found on the same surface. Such stimuli with conflicting gloss information across the surface could be used to investigate to what extent the visual system relies on local or global cues for glossiness and how the gloss impression is affected by local irregularities of the highlight features. Another way to produce highlight patterns with different degrees of overlap was suggested by one of the reviewers: One could use surfaces that contain only a few locations of extreme curvature. For certain smoothness levels, the highlights of a group will appear as merged on some locations and separated at others.

## Conclusions

We investigated how gradual changes in the distance between three point light sources (“light spread”) influence the perceived glossiness of objects in the illuminated scene. Our results indicate that this manipulation often significantly impairs gloss constancy. The main cause seems to be that highlights related to different light sources overlap to such a degree that they cannot be discerned by the visual system and are misinterpreted as a single more extended highlight. This then leads to an underestimation of surface glossiness. The degree of overlap depends both on surface smoothness, which influences the width of individual highlights, and the distance between the light sources, which influence their spatial separation. If smoothness and spread were chosen in such way that overlaps of highlights were small or absent, gloss constancy across conditions that differed considerably with respect to the number and intensity of highlights was substantially improved. This finding seems to indicate that the visual system relies mainly on local highlight features and much less on global image cues when judging the glossiness of a surface. However, we found that a large part of the variance in the perceived glossiness of our stimuli could be explained by a linear model that is based on a set of different global image cues, like the relative size of the highlight area and its mean intensity.

## Figures and Tables

**Figure C1.**
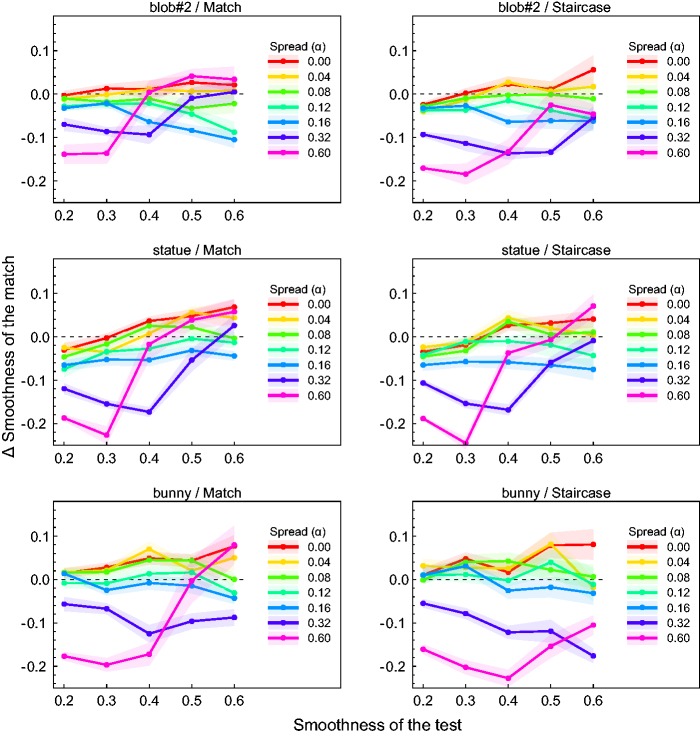
Results of the preexperiment, averaged across all three subjects. The diagrams are ordered by shape condition (rows) and the psychophysical method (columns). The original smoothness settings were transformed into the measure Δ smoothness. The mean Δ smoothness values are plotted against the smoothness of the test, with light spread as grouping variable (colored lines). Transparent areas around each curve represent ±*SEM*.

**Figure E1.**
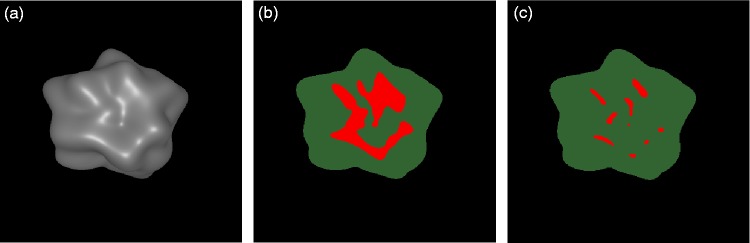
(a) Example of a test stimulus (here one half-image of the stereo pair). (b) The respective matching stimulus with the biggest possible extension of the highlight pattern (red areas). (c) Example matching result for the perceived sizes of the highlights in (a).

## Appendix A—Linearized Smoothness Scale

In Experiment 1, the subjects matched glossiness by adjusting surface smoothness. To facilitate this task, we conducted a scaling experiment to determine an approximately linear smoothness scale with the property that equal changes in smoothness correspond to equal changes in perceived glossiness. To this end, Maximum Likelihood Difference Scaling (MLDS) as proposed by Maloney and Yang (2003) was used. In each trial, the subject compared a Pair A of stimuli with a second Pair B. The stimuli in each pair were illuminated surfaces with different “original” smoothness values as used in Unity’s standard shader. The subject then decided, whether Pair A differed more in glossiness than pair B. Based on the set of inequalities produced in this way, the functional relationship between gloss and smoothness was determined and a linear scale constructed.

The shape blob#2 depicted in Figure 5 was the test object. During the presentation, it rotated counterclockwise around its vertical middle axis at a speed of 60°/s. A single point light source located in front of the object was used (see the central light in Figure 4). The intensity level of the light was set to 1.5. Eleven different values of the original smoothness scale were used (0 to 0.9 in steps of 0.1 plus the value 0.95; larger values were omitted, because these correspond closely to a perfect mirror and thus lead to nearly point-like highlights). We adopted the method of nonoverlapping quadruples as described in Knoblauch and Maloney (2008). For the 11 smoothness levels, this leads to a total number of 330 quadruples of stimuli which were presented in random order during the experiment.

The stimuli were presented monocularly, one pair above the other for at least 5 seconds. After this time period, the subject was able to make his judgment, using the up and down arrow keys of the keyboard. The next trial started after a short adaptation interval of 1 second during which the entire screen appeared in the blueish background color. The subject was one of the authors (G. W.).

The MLDS package for R (Knoblauch & Maloney, 2008) was used to evaluate the data. A fit of a power function to the resulting scaling curve revealed an exponent of 1.77. This indicates that the ability to discriminate between different degrees of glossiness increases with higher smoothness values. The linear scale is thus defined as scaled smoothness = original smoothness^1/1.77^. If not stated otherwise, the term smoothness in the text always refers to this scaled smoothness parameter.

### Appendix B—Effective Illumination Color of a Point Light in Unity

The point lights in Experiment 1 had a maximum effective range *r_max_* of 10 units. Within this range, the light intensity of a point light drops as a function of the distance *r* (with *0 ≤ r ≤ r_max_*) according to Equation (1):

(1) $$\mathrm{att}=1.0/(1.0+25.0\times (r/r_{\text{max}}{)}^{2})$$

In order to calculate the illumination color *Ie* that directly affects a surface point on an object, the original illumination color *Io* (which was rgb = (1.0, 1.0, 1.0) in Experiment 1) is multiplied by this attenuation factor and by the respective light intensity *i* that can take values between 0 and 8 (in Experiment 1 we used either 0.5 or 1.5 as the intensity for the point lights):

(2) $$\begin{array}{c} (\begin{array}{c} {\mathrm{Ie}}_{r}\\ {\mathrm{Ie}}_{g}\\ {\mathrm{Ie}}_{b} \end{array})=\mathrm{atti}(\begin{array}{c} {\mathrm{Io}}_{r}\\ {\mathrm{Io}}_{g}\\ {\mathrm{Io}}_{b} \end{array}) \end{array}$$

The resulting effective illumination color is then used to calculate the actual material color of a point on an object’s surface according to the BRDF model. The respective shader files can be obtained at Unity’s download archive page (unity3d.com/get-unity/download/archive).

### Appendix C—Comparison of Staircase and Matching Procedures

Previous studies have shown that judgments of surface material can depend on the psychophysical method used in the measurement (Doerschner et al., 2010, p. 4). To explore potential effects of the task on measured glossiness, we compared in a preexperiment glossiness judgments obtained from pair comparisons in a staircase procedure with those obtained using a matching task. Possible effects of task on gloss perception could be caused by different perceptual sets: Presumably, pair comparisons are more based on the global impression, whereas the matching task prompts a focus on local features.

The general set-up of the matching experiment was identical to that described in Experiment 1, but only three test objects (blob#2, statue, and bunny, see Figure 5) and only one intensity level for the point light sources of the test (0.5, see section Lighting) were used. Four repetitions per condition led to a total of 420 trials (3 Shapes × 1 Light Intensity × 7 Light Spreads × 5 Smoothnesses × 4 Repetitions) which were presented in random order during the experiment. The same set of stimuli was used for the pair comparison task. In this experiment, we used a two-alternative forced choice paradigm, implemented in a double random staircase procedure (Cornsweet, 1962). The comparison stimulus, which was always presented on the top half of the monitor, was identical to the matching stimulus in the matching task, with the exception that its smoothness parameter was varied according to the following rules during one trial (where each trial consisted of up to 50 steps): In each trial, the two staircases S_A_ and S_B_ started at opposite ends of the smoothness scale, S_A_ at a scaled smoothness value of 0.1 and S_B_ at 0.9. One of the two staircases was randomly chosen as the active one. The starting value for the step size for the smoothness was 0.3 for both staircases. At each step, the subject had to indicate whether the comparison or the test stimulus appeared glossier, using the up or down arrow key of the keyboard. If the comparison stimulus was judged as glossier, then the current parameter value was decreased by the current step size, otherwise it was increased accordingly. Whenever a change in direction occurred during one of the staircases, that is, whenever a test stimulus that formerly appeared less glossy (glossier, respectively) compared to the comparison stimulus, started to appear glossier (less glossy, respectively), the step size was divided by two. A trial ended when each of the two staircases had at least seven changes, or when the total number of steps exceeded 50. The result of the trial was the mean of the smoothness values of the comparison stimulus over the last six steps. Three subjects participated in this experiment, including one of the authors (G. W.).

Figure C1 shows the results averaged across all subjects: The diagrams in the left column show the results for the matching task, the ones in the right column those for the pair comparison task. The single diagrams of each column show the results for the three different shapes. We transformed the original smoothness settings into the measure “Δ smoothness” by subtracting the true smoothness of the respective test stimulus. In each diagram, the mean Δ smoothness is plotted against the smoothness of the test with light spread as grouping variable (differently colored lines in Figure C1).

It can be seen that perceived glossiness depends strongly on both the light spread and the smoothness of the test. Our interpretation of the complex pattern is discussed in the results section of Experiment 1. Here, we will focus on the question whether the glossiness settings are systematically influenced by the experimental method. An analysis of variance actually yielded a highly significant main effect of the psychophysical method (with *p* < .001). On closer inspection, one can see that occasionally corresponding data points measured with both methods deviate considerably from each other, for instance in the case of the bunny object (third row in Figure C1) when high smoothness values are combined with the highest light spread (pink lines). The exact causes of these differences are at present unknown and could be the subject of further investigations. Nevertheless, the general trend in the data seemed similar enough to justify the use of the much more economic matching task in Experiment 1 (the pair comparison task took about 8 times longer).

### Appendix D—ANOVA Results from Experiment 1

Table D1 shows the results of the four-way ANOVA that was performed on the data of Experiment 1 with the factors shape, smoothness, light spread (“Spread”), and light intensity (“Intensity”). All four main effects as well as all first order interaction effects were considered. With the exception of the interaction between shape and light intensity, all of these effects were statistically significant.

**Table D1.** Results of the ANOVA Performed for Experiment 1.

Source	Sum Sq.	*df*	Mean Sq.	*F*	*p*
Shape	9.346	4	2.3365	328.91	<.001
Smoothness	132.519	4	33.1298	4663.76	<.001
Spread	4.368	6	0.728	102.48	<.001
Intensity	8.369	1	8.3692	1178.16	<.001
Shape × Smoothness	0.551	16	0.0344	4.84	<.001
Shape × Spread	1.025	24	0.0427	6.01	<.001
Shape × Intensity	0.041	4	0.0102	1.43	.2209
Smoothness × Spread	2.589	24	0.1079	15.19	<.001
Smoothness × Intensity	0.696	4	0.174	24.5	<.001
Spread × Intensity	0.11	6	0.0184	2.59	.0165
Error	49.058	6906	0.0071		
Total	208.672	6999			

### Appendix E—Matching of Perceived Highlight Areas

Qi et al. (2015) used a simple fixed criterion to isolate the highlight regions in a stimulus. As an alternative, we determined the perceived gloss layer in an empirical way. To this end, the subject was asked to interactively adjust the size of a pattern of homogeneously colored patches in a comparison stimulus such that it matched the perceived extensions of the highlights in the test stimulus.

The test stimuli were the same 350 pictures that were used in Section “Test of Global Glossiness Cues” to determine the image statistics proposed by Qi et al. (2015).

For the construction of the matching stimulus, we used a modified version of the test image in each trial. As a preparatory step, we additionally rendered a diffuse image *I_d_* for each of the 70 different combinations of shape, light spread, and intensity, that is, the object had a smoothness parameter value of 0 in these cases. This diffuse image *I_d_* was used to extract the objective gloss layer *I_g_* from the original image *I_o_* by pixelwise subtracting *I_d_* from *I_o_* (*I_g__ _= I_o_ – I_d_*). The resulting gloss image *I_g_* represented the highlight pattern of the original image in its true extension which, at least for our stimuli, was always considerably larger than the perceived extensions of the highlights (see Figure E1). To alter the size of the highlights in the pattern during the experiment, the subject interactively adjusted the pixel intensity threshold of the gloss image within a range between 0 and max(*I_g_*). Pixels in the gloss image that exceeded this intensity threshold were set to a reddish color in the matching stimulus (rgb = 0.977, 0, 0) while the rest of the stimulus, that is, the diffuse part, was set to a uniform greenish color (rgb = 0.194, 0.391, 0.194) such that the contour was identical to that of the object in the respective test stimulus (see Figure E1).

Hence, a pixel intensity threshold of 0 produced a highlight pattern with maximum extension and a pixel intensity threshold of max(*I_g_*) led to a matching stimulus that had no highlights (i.e., no reddish pixels) at all.

The task of the subject was to adjust the size of the reddish patches in the matching stimulus such that their extensions equaled those of the perceived highlights in the test stimulus, using the left and right arrow keys of the keyboard. However, in some cases such a perfect match was hard to achieve: It could happen that when the size of a certain patch seemed to match that of its corresponding highlight in the test another patch seemed to differ from its counterpart to some degree, and vice versa. Therefore, the subject was instructed to achieve the best match possible on an individual highlight basis while additionally taking care to also match the total extension of the entire highlight pattern, that is, to match the overall coverage (or percentage highlight area) between the two stimuli.

After each matching trial, the subject was asked to judge how satisfied he was with the matching result on an individual highlight basis, that is, how good the matched sizes of the single patches in the matching stimulus corresponded to the perceived sizes of the respective highlights in the test. We used a rating scale between 0 and 5 where a value of 0 meant that the sizes vastly differed between the two stimuli and a value of 5 represented a perfect match.

The test image was always presented in the top half of the monitor, the matching stimulus always in the bottom half. The rating was done after the match while the two stimuli were still visible. Then a dark adaptation period of 3 seconds followed after which the next trial started. We used the same apparatus and viewing conditions as in Experiment 1, that is, both stimuli were presented stereoscopically by means of a mirror stereoscope. We tested each of our 350 stimuli 3 times, and the entire set of 1,050 trials was presented in random order. There were no time limits to the task and a session could be interrupted at any time and later resumed. The subject was one of the authors of the present article (G. W.).

The following rule was applied to determine the mean intensity threshold from the three settings made by the subject: Only settings that produced gloss layers with a positive number of reddish pixels were considered as “valid.” This means that settings indicating the absence of a gloss layer (with no red pixels) were excluded from the calculation. A mean threshold value was only calculated for stimuli with at least two valid settings. Otherwise, this stimulus was marked as an invalid case and was not taken into account for any further data analysis (see Figures 13 and 14).

A brief look at the rating data seems to suggest that in general our matching experiment was a manageable task for the subject: None of the matches was rated as 0 and the average rating value across all trials was 2.87. However, the degree of satisfaction in the matching task shows a strong dependence on the stimulus conditions. We performed a four-way ANOVA on our rating data and found significant main effects for the shape, *F*(4,345) = 72.06, *p* < .001, the smoothness, *F*(4,345) = 15.64, p < .001, and the light spread factor, *F*(6,343) = 5.09, *p* < .001. Most notable is the influence of shape on the rating values: According to a Bonferroni post hoc test, the matches obtained under the shape condition “blob#1” (with an average rating value of 2.00 across the entire data set) were rated significantly lower than each of the other shape conditions which had average values between 2.89 and 3.34.

The pixel intensity threshold settings of the subject were later used to estimate the perceived gloss layer for each stimulus, which in turn was used to calculate the image statistics proposed by Qi et al. (2015). These results are presented and discussed in Section “Test of Global Glossiness Cues”.
